# Experiences of Prisoners Living With Mental Health Conditions and Seeking or Receiving Mental Health Support in Prisons: An Integrative Review

**DOI:** 10.1002/cbm.70046

**Published:** 2026-07-24

**Authors:** Anne Kairu, Judith Dyson, Damian Breen, James Skinner, Manuela Jarrett, Joanne Brooke

**Affiliations:** ^1^ School of Nursing and Midwifery Birmingham City University Birmingham UK; ^2^ School of Law and Social Sciences Birmingham City University Birmingham UK; ^3^ Department of Criminology Sociology and Social Policy School of Social Sciences Swansea University Swansea UK; ^4^ Department of Nursing and Midwifery College of Medicine and Health University of Birmingham Birmingham UK

## Abstract

**Background:**

Prisoners experience poor mental health and well‐being in prison, including severe mental illnesses. However, access to mental health support and treatment in prison is limited. We conducted an integrative review to synthesise prisoners' experiences of living with mental ill health and/or seeking or receiving mental health support in prisons.

**Methods:**

A systematic search of peer‐reviewed empirical studies published from 2014 to 2024 was conducted following PRISMA guidelines. Databases searched included Medline, PsycINFO and CINAHL. We included studies of adult prisoners (aged ≥ 18 years) experiencing poor mental health who sought or received help. We were inclusive in our definitions of “poor mental health” and “support” to capture papers where prisoners may not have a formal diagnosis and where support may have been pastoral or delivered by healthcare practitioners.

**Results:**

Nineteen studies met the inclusion criteria. Four main themes emerged. The first encompassed the range of emotions and feelings experienced, predominantly negative. The second was about the accessibility of mental health services, evidenced not only by limited availability but also by a general lack of support, service providers being described as lacking empathy and coercion to take medication. The third theme was of more internalised barriers to help‐seeking, including a sense of stigma and concerns about confidentiality. The fourth theme was about coping strategies, including support from peers, family and religious practices but also maladaptive coping strategies such as self‐harm.

**Conclusions:**

Prisoners experienced unmet mental health needs. Poor service experiences added to the burden, only partly met through constructive alternatives. Serious, sometimes dangerous maladaptive coping suggests that current access to and suitability of formal healthcare services should be reconsidered within the context of wider methods of pastoral support. Future research on the role of the wider prison community in providing mental health and well‐being support is needed.

**Trial Registration:**

The protocol for this review is registered on PROSPERO CRD42024581279. Available from https://www.crd.york.ac.uk/prospero/display_record.php?ID=CRD42024581279

## 1 Introduction

The global prison population is rising (Forrester et al. 2018), with mental health problems affecting up to 90% of the prison population (over 80,000 prisoners) in England and Wales (Durcan 2023). Internationally, mental ill health rates are higher for people in prison than in the community (Baranyi et al. 2022; Fazel et al. 2016). In this paper, we define mental ill health to include “disorders”, or clinically significant behavioural or psychological patterns associated with painful symptoms, for example, distress or impairment (Telles‐Correia et al. 2018). We also include feelings of distress caused by trauma (Karlsson and Zielinski 2020) in our definition of mental ill health. The prison population is one with high levels of childhood adversity (Bowen et al. 2018), low educational attainment levels (Ministry of Justice 2024) and most prisoners coming from economically deprived communities (Pavicevic and Ilijic 2022). People from deprived areas in England are 10 times more likely to be in prison than those from more affluent areas (Hart et al. 2022). Prison safety statistics from England and Wales reported 96 self‐inflicted deaths between September 2024 and 2025 and 76,148 self‐harm incidents between June 2024 and 2025, a rate of 878 self‐harm incidents per 1000 prisoners in this one year (Ministry of Justice 2025).

Prison environments exacerbate these vulnerabilities. Environmental stressors such as prolonged isolation (Zhong et al. 2021), overcrowding and restrictive practices (Fazel et al. 2016) have been identified as contributors to prisoners' mental health decline. A recent systematic review of 24 studies and 23,967 prisoners reported prison‐based victimisation as a global public health problem, being five to six times higher than in the community (Caravaca‐Sánchez et al. 2023). Over 18 per cent of men and 20% of women prisoners experience physical victimisation, with sexual victimisation being experienced by 9.7% and 15.3%, respectively (Caravaca‐Sánchez et al. 2023).

Despite high rates of mental ill health and the stressors associated with the prison environment, 49% of UK prisons report mental health staff shortages (Durcan 2023). Mental health treatment needs are unmet due to resource constraints and security concerns (Woodall and Freeman 2021). Limited healthcare resources within the prison system mean prisoners' health and well‐being needs are unlikely to be met (Franke et al. 2019). Other support initiatives are ad hoc and dependent on available funding and resources (Turner et al. 2022). Our aim, therefore, was to conduct an integrative review to synthesise global peer‐reviewed empirical literature on prisoners' experiences of mental health and support services.

## 2 Methods

Our review question was as follows: “What are prisoners experiences of living with mental health conditions and/or seeking or receiving mental health support in prisons?”

### 2.1 Design

This review followed the guidelines outlined in the Preferred Reporting Items for Systematic reviews and Meta‐Analyses (PRISMA) (Page et al. 2021).

#### 2.1.1 Search Strategy and Selection Criteria

A comprehensive literature search was conducted in electronic databases, including Medline, PsycINFO and CINAHL. The search was limited to studies published in English from 2014 to 2024, with data collected from 2011 (Ferrari 2015). A preliminary search, including on PROSPERO, ensured no other related systematic reviews had been conducted or were currently being conducted. The protocol for this review has been published (PROSPERO registration CRD42024581279, 2024). Forward and backward citation tracking of retrieved articles was conducted by manually searching reference lists to maximise the number of relevant papers. With the support of a subject specialist librarian, the search strategy was adapted for each database; however, the following string summarises the terms, Boolean operators and truncation used.

Prisoner* OR inmate* OR imprison* OR convict* OR offender* OR detainee* OR incarcerate*

AND

“Mental health” OR “mental illness*” OR “mental disorder*” OR “mental health problem*” OR “mental ill‐health” OR “mental ill$health” OR “psychiatric problem*” OR “psychiatric disorder*” OR “psychiatric illness*” OR “psychological problem*” OR “psychological disorder*” OR “psychological issue*” OR “psychological illness*”

AND

Experience* OR perception* OR opinion* OR attitude* OR view* OR feeling*

Inclusion criteria were empirical, peer‐reviewed studies to ensure methodological rigour. We included studies published in English between January 2014 and November 2024 to ensure comprehensive evidence that was relevant to current prison mental health practices and experiences. We captured studies that investigated adult (aged ≥ 18 years) prisoners' experiences of living with mental health conditions and/or seeking or receiving mental health support in prisons, whether sentenced prisoners or those in pretrial detention. Included studies explored experiences related to living with mental illness in the prison population, including support mechanisms, risk factors, availability and accessibility of mental health services and coping strategies. Studies were excluded if they focused on prison staff, younger prisoners (under 18 years), individuals in forensic psychiatric wards, immigration detention, military settings and former prisoners or those who accessed support outside prison.

#### 2.1.2 Selection Process

After the search was undertaken, the studies were downloaded to Rayyan (*Rayyan: a web and mobile app for systematic reviews* 2016), and duplicates were removed. Titles, abstracts and full texts were independently screened by at least three reviewers, and this was followed by discussions with all the authors. Figure 1 shows the PRISMA flow diagram (Page et al. 2021) summarising the study selection process at each stage.

**FIGURE 1:**
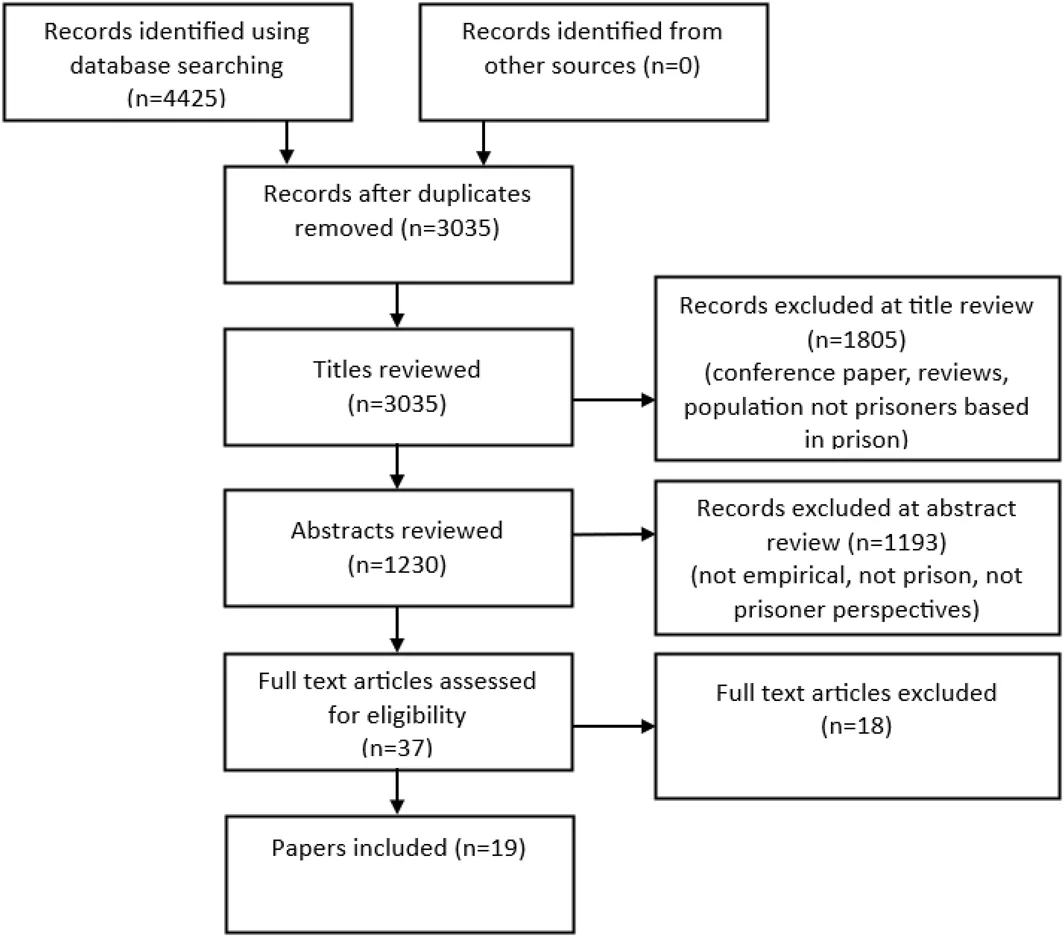
PRISMA flow diagram.

#### 2.1.3 Quality Assessment

The quality of the included studies was evaluated using the relevant Critical Appraisal Skills Programme (CASP) checklist (CASP 2025) or the critical appraisal of cross‐sectional study (Center for Evidence‐Based Management 2014). Each study was critically evaluated against these criteria, and deviations from quality standards were systematically recorded and analysed. All studies were included irrespective of quality.

#### 2.1.4 Data Extraction and Analysis

Data were extracted using a custom‐designed spreadsheet, which recorded the study's aims, participants, settings, methods and outcomes (see Table 1). Information related to prisoners' experiences of living with mental ill health, seeking and/or receiving mental health support, the barriers to seeking and/or receiving support and coping mechanisms used by prisoners were extracted. The extraction process also recorded survey data on any of these factors and self‐reported mental health experiences and conditions reported across the included studies. Where there was sufficient homogeneity, we synthesised survey data using frequencies and percentages; otherwise, narrative synthesis was used to combine the data according to the review's focus through data visualisation, description and interpretation (Ferrari 2015).

**TABLE 1:** Summary of findings.

First author year	Participants, country (number)	Aim	Methods	Results in relation to research question	Quality assessment score and exceptions
Antonetti 2018 (Antonetti et al. 2018)	Female prisoners, Italy (*n* = 55)	Gather information about the needs of women in prison	Survey	Dissatisfaction with the mental health support provided. Psychological needs were unmet. Lack of positive relationships and trust in the prison environment. Coping strategies included high levels of smoking and substance abuse.	10/12, participant recruitment strategy unclear, sample size justification unclear
Bentley 2017 (Bentley and Casey 2017)	Female prisoners, USA (*n* = 274)	Investigate beliefs about psychotropic medication	Survey	77% of prisoners took psychotropic medication, mainly antidepressants. The main barrier to seeking/receiving support was stigma related to medication use.	11/12, unclear whether the response rate was satisfactory
Cobb 2014 (Cobb and Farrants 2014)	Male prisoners, England and Wales (*n* = 9)	Identify male prisoners' help‐seeking behaviour	Semi‐structured interviews	Barriers to seeking help were medication being seen as a tool to control rather than rehabilitate, perceived injustice and disrespect in the system and coercion with medication. Help‐seeking was perceived as a weakness and stigmatised as a way to ‘work the system’.	9/10, relationship between researcher and participants not reported
Constantino 2016 (Constantino et al. 2016)	Male and female prisoners, Brazil (*n* = 1573)	Examine the association between mental health and imprisonment	Survey	35.8% of men and 57.9% of women experienced stress due to the length of time in prison and family ties. 7.5% of women and 6.3% of men were depressed. Maintaining family ties and performing prison work tasks were protective factors.	11/12, response rate unclear
Costa 2021 (Costa et al. 2021)	Male prisoners, Europe (*n* = 483)	Compare prison staff and prisoners' perceptions of mental health support and access	Survey	Prisoners preferred self‐reliance or support from family rather than formal prison support structures. They did not know whom to turn to for help and faced stigma, lack of trust and limited access to timely services.	11/12, response rate unclear
Clemente‐Faustino 2022 (Clemente‐Faustino and de Guzman 2022)	Male detainees, Philippines (n‐11)	Explore self‐harming behaviour experiences	Semi‐structured interviews	Stress, overcrowding and lack of family visits contributed to deteriorating mental health. Adherence to jail rules was a coping mechanism. Self‐harming behaviours were common as a response to distress.	9/10, relationship between researcher and participants not reported
Fedock 2024 (Fedock et al. 2024)	Female prisoners, United States (*n* = 42)	Explore women prisoners' agency in mental health treatment	Semi‐structured interviews	Prisoners reported that mental health treatment lacked adequate follow‐up, there was inadequate staff training and were short appointments. Women were concerned about confidentiality and lack of privacy. Creative outlets such as painting and journalling relieved symptoms.	9/10, relationship between researcher and participants not reported
Hassan 2015 (Hassan et al. 2015)	Male and female prisoners, England and Wales (*n* = 17)	Analyse perspectives on psychotropic medication changes in prisons	Semi‐structured interviews	Prisoners reported short appointments and abrupt medication changes, without consultation, causing distress, confusion and causing mood swings, insomnia and self‐harm. Perceived lack of genuine staff care. Distrust in mental health professionals and the prison doctor.	9/10, relationship between researcher and participants not reported
Hemming 2023 (Hemming et al. 2023)	Male prisoners, England) (*n* = 9)	Explore male prisoners' emotions before suicide and acts of violence	Interviews and participatory method (drawing emotions)	Emotions included despair, hopelessness, isolation, fear, anger and sadness. The impact of past trauma was highlighted.	9/10, relationship between researcher and participants not reported
Maruca 2017 (Maruca et al. 2017)	Male (*n* = 35), female (*n* = 20) and other prisoners (*n* = 1), United States	Explore self‐care perceptions among prisoners	Focus groups	Coping strategies included reading, avoiding negativity and staying in therapy.	9/10, relationship between researcher and participants not reported
Milavetz 2021 (Milavetz et al. 2021)	Parents in prison, USA (*n* = 165)	Investigate mental health and support needs for jailed parents with young children	Interviews and survey	Depression and thought problems were identified, exacerbated by childhood trauma and parenting stress. Gender differences were evident, with mothers reporting more issues than fathers. Only a small proportion received mental health treatment.	Survey, 11/12, no pre‐study power calculation mentioned. Interviews, 10/10
Nwefoh 2020 (Nwefoh et al. 2020)	Male prisoners, Nigeria (*n* = 381)	Investigate the psychosocial consequences of remand	Survey	A high prevalence of depression (37.8%) and a lack of detection and treatment for mental disorders were reported.	11/12, response rate unclear
Rose 2020 (Rose and LeBel 2020)	Pregnant female prisoners, USA (*n* = 27)	Examine the health challenges of pregnant women in jail	Survey	48.2% received mental health treatment and 18.5% received treatment for substance use, though, 55.6% screened positive for substance use.	11/12, response rate unclear
Runte‐Geidel 2014 (Runte‐Geidel et al. 2014)	Male prisoners, Spain (*n* = 6)	To ascertain opinions on coercive measures	Focus group	Prisoners felt overmedicated and poorly informed about their conditions. Coercive measures and forced medication were experienced.	8/10, recruitment strategy and relationship between researcher and participants unclear
Solbakken 2023 (Solbakken et al. 2023)	Male prisoners, Norway (*n* = 15)	Explore mental health and coping in prison	In‐depth interviews	Prisoners expressed despair and hopelessness, stress and anxiety, were reluctant to take medication and coped through social interactions and activities.	9/10, relationship between researcher and participants unclear
Vilhjalmsdottir 2023 (Vilhjalmsdottir et al. 2023)	Female prisoners, Iceland (*n* = 9)	Explore women's experiences of prison, trauma and substance use	In‐depth interviews	Participants expressed despair and hopelessness, stress and anxiety, pre‐prison trauma and used substances to cope.	9/10, relationship between researcher and participants unclear
Walker 2017 (T. Walker, Shaw, et al. 2017a)	Female prisoners, England (*n* = 13)	Explore the impact of good‐bye letters and after psychodynamic interpersonal therapy	Semi‐structured interviews	Participants considered the letters a form of support, rereading the letter when needed and sharing them with others as a means of connection.	9/10, relationship between researcher and participants unclear
Walker 2021 (T. Walker et al. 2021)	Female prisoners, England and Wales (*n* = 108)	Determine patterns, prevalence and functions of self‐harm among imprisoned women	Interviews and survey	High levels of emotional distress were linked to self‐harm. Trauma sometimes stemmed from domestic violence and childhood abuse. Support was available from a safer custody team, but medical support was limited. Self‐harm was used to manage emotions.	Survey, 11/12, no pre‐study power calculation mentioned. Interviews, 10/10
Western 2021 (Western et al. 2021)	Male prisoners, USA (*n* = 99)	Explore the harms associated with solitary confinement	Survey and interviews	Psychological distress was experienced in solitary confinement due to inactivity, social isolation and feelings of dehumanisation. Access to mental health support was limited.	Survey, 11/12, selection bias (acknowledged). Interviews, 10/10

## 3 Results

Table 1 provides a summary of included studies, including exceptions to quality. Table 2 indicates the disorder context in those studies that were large enough and had sufficient homogeneity to synthesise survey data in terms of frequencies and percentages.

**TABLE 2:** Range of mental health problems recorded after pooling available survey data from included studies.

Mental health problem	First author and year								Mean % (total number)
	Antonetti et al. (2018)	Bentley and Casey (2017)	Constantino et al. (2016)	Milavetz et al. (2021)	Nwefoh et al. (2020)	Rose and LeBel (2020)	T. Walker et al. (2021)		
Psychological disorders	65.5% (*n* = 36)								65.5% (*n* = 36)
Self‐harm							52% (*n* = 105) Emotional relief	12.4% (*n* = 25) Control	32.1
Anxiety		76% (*n* = 209)	42.3% (*n* = 665)	9% (*n* = 15)		22.2% (*n* = 6)			37.4% (*n* = 895)
Depression		72% (*n* = 196)	29.1% (*n* = 458)	16% (*n* = 26)	37.8% (*n* = 144)	40.7% (*n* = 11)			
Psychotic disorder		10% (*n* = 26)				11.1% (*n* = 3)			10.55% (*n* = 29)
Bipolar disorder		47% (*n* = 128)				7.4% (*n* = 8)			27.2% (*n* = 136)

### 3.1 Quality Appraisal

Exceptions to quality were recorded (see Table 1). In summary, exceptions to quality tended to be unclear reporting on the relationship between researchers and participants, and recruitment strategies were not always replicable.

### 3.2 Characteristics of Included Studies

Nineteen studies met the criteria for inclusion in this review. One study was conducted across five European countries (Costa et al. 2021) and one in each of the following countries: Brazil (Constantino et al. 2016), Iceland (Vilhjalmsdottir et al. 2023), Italy (Antonetti et al. 2018), Nigeria (Nwefoh et al. 2020), Norway (Solbakken et al. 2023), the Philippines (Clemente‐Faustino and de Guzman 2022) and Spain (Runte‐Geidel et al. 2014). Five studies were conducted in the United Kingdom (Cobb and Farrants 2014; Hassan et al. 2015; Hemming et al. 2023; T. Walker et al. 2021, 2017a) and six in the USA (Bentley and Casey 2017; Fedock et al. 2024; Maruca et al. 2017; Milavetz et al. 2021; Rose and LeBel 2020; Western et al. 2021).

Various study designs were used, including focus groups (Maruca et al. 2017), semi‐structured interviews (Clemente‐Faustino and de Guzman 2022; Cobb and Farrants 2014; Fedock et al. 2024; Hassan et al. 2015; Solbakken et al. 2023; Vilhjalmsdottir et al. 2023; T. Walker, Shaw, et al. 2017a), surveys (Antonetti et al. 2018; Bentley and Casey 2017; Constantino et al. 2016; Costa et al. 2021; Nwefoh et al. 2020; Rose and LeBel 2020; Western et al. 2021) and mixed methods (Milavetz et al. 2021; T. Walker et al. 2021; Western et al. 2021). All included studies pertained to prisoners' experiences of mental health and illnesses and the help provided through varied approaches. Some survey‐based studies included diagnosed mental illnesses (Bentley and Casey 2017). Others used assessment tools, including the Patient Health Questionnaire‐9 (PHQ‐9) for depression screening (Nwefoh et al. 2020) and the Lipp Inventory of Stress Symptoms for Adults (Constantino et al. 2016). Other survey studies gathered data through self‐reported responses to structured questionnaires (Antonetti et al. 2018; Milavetz et al. 2021; Rose and LeBel 2020; T. Walker et al. 2021). Although most interview‐based studies used semi‐structured interviews, one study integrated a participatory visual method, which involved drawing emotions to help overcome verbal expression barriers (Hemming et al. 2023). One study using focus groups incorporated prison staff, but only data from the prisoners were analysed to maintain focus on prisoner experiences (Runte‐Geidel et al. 2014).

The synthesis included 3465 participants. Sampling diversity was evident, including male, female and other participants (Maruca et al. 2017); jailed parents with young children (Milavetz et al. 2021); pregnant women (Rose and LeBel 2020); mixed‐gender groups of males and females (Constantino et al. 2016; Hassan et al. 2015); males (Clemente‐Faustino and de Guzman 2022; Cobb and Farrants 2014; Costa et al. 2021; Fedock et al. 2024; Hemming et al. 2023; Nwefoh et al. 2020; Runte‐Geidel et al. 2014; Solbakken et al. 2023; Western et al. 2021); and females (Antonetti et al. 2018; Bentley and Casey 2017; Fedock et al. 2024; Vilhjalmsdottir et al. 2023; T. Walker et al. 2021, 2017a, 2017b). The studies represented a variety of racial and ethnic backgrounds including Asian, Black, Hispanic, White, American first Nation people/Alaska Native people (Maruca et al. 2017), and African American and Caucasian (Milavetz et al. 2021). One study in the USA reported mental health conditions and experiences including 48% White, 40.5% Black/African American, 9.5% Latine and 2% Asian American participants (Fedock et al. 2024). The data from all included studies were blended in the narrative review; demographic characteristics (gender and ethnicity) were noted as reported in the original studies but were not independently analysed or compared within this review.

### 3.3 Findings

Four core categories of experience were identified: (1) emotions and feelings, mainly negative; (2) accessibility of mental health services, mainly encompassing difficulties with access; (3) barriers within the self to seeking or receiving support, however triggered; and (4) coping strategies. All categories and subcategories are presented in Figure 2 and discussed in the narrative that follows.

**FIGURE 2:**
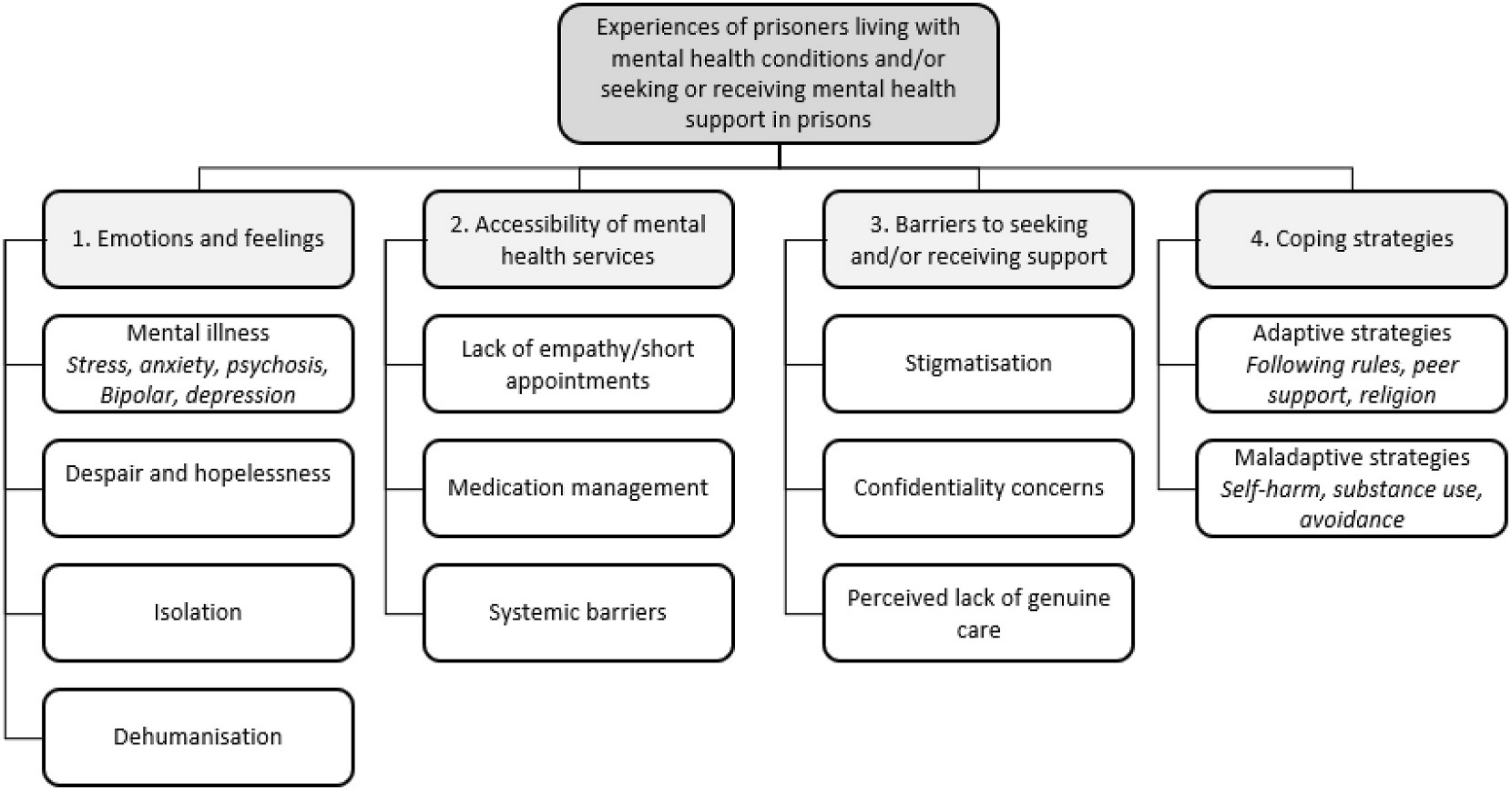
Themes and subthemes.

#### 3.3.1 Emotions and Feelings

This theme of emotions and feelings included specific mental health conditions, despair and hopelessness, isolation and dehumanisation.

##### 3.3.1.1 Mental Illness

Mental illness experiences, as reported across these studies, broadly fit with data from primarily prevalence studies. They included bipolar disorder (Bentley and Casey 2017; Maruca et al. 2017; Rose and LeBel 2020; Runte‐Geidel et al. 2014; Western et al. 2021), post‐traumatic stress disorder (PTSD) (Maruca et al. 2017), substance abuse (Maruca et al. 2017; Rose and LeBel 2020), attention deficit hyperactivity disorder (ADHD) (Milavetz et al. 2021), psychological disorders (Antonetti et al. 2018), depression (Maruca et al. 2017; Milavetz et al. 2021), anxiety (Bentley and Casey 2017; Constantino et al. 2016; Maruca et al. 2017; Milavetz et al. 2021; Rose and LeBel 2020), borderline personality disorder (Runte‐Geidel et al. 2014), schizophrenia (Runte‐Geidel et al. 2014; Western et al. 2021), psychotic disorder (Bentley and Casey 2017; Rose and LeBel 2020) and self‐harm (T. Walker et al. 2021). Survey data revealed homogeneity across seven studies (Antonetti et al. 2018; Bentley and Casey 2017; Constantino et al. 2016; Milavetz et al. 2021; Nwefoh et al. 2020; Rose and LeBel 2020; T. Walker et al. 2021). Psychological disorders were reported in about two thirds of prisoners (Antonetti et al. 2018), and self‐harm reports ranged from just over a fifth of prisoners who engaged in self‐harm for control purposes to just over half of those who used self‐harm for emotional relief (T. Walker et al. 2021).

Overall, more than a third of all participants reported anxiety, with a range from 9% (Milavetz et al. 2021) to 76% (Bentley and Casey 2017). As anxiety was not formally rated in all studies, these summary figures are likely to be underestimates. Factors associated with stress among men included the length of time in prison and the state of family ties, with men imprisoned for less than a year being more likely to experience stress than those imprisoned for longer (Constantino et al. 2016). Prisoners described a cyclical nature of negative emotions where stress and anxiety fed into a loop of anger and sadness (Solbakken et al. 2023). Overall, rates of depression across the five studies, with a combined sample of 835 prisoners, ranged from 16% (Milavetz et al. 2021) to 72% (Bentley and Casey 2017). There were no data in any of the qualitative studies that explained this further. There was little variation in the reporting of psychosis, with estimates ranging from 10% (Bentley and Casey 2017) to 11% (Rose and LeBel 2020); bipolar disorder prevalence in studies reporting it was a bit more variable, with just over a quarter of those participants asked reporting it, but a range from just over 7% (Rose and LeBel 2020) to 47% (Bentley and Casey 2017). A lack of detection and treatment for mental disorders was reported, with one Nigerian study noting that very few prisoners were receiving psychotropic medications or counselling (Nwefoh et al. 2020).

##### 3.3.1.2 Despair and Hopelessness

Women struggled with complex mental health problems after severe traumas, including violence, bullying and neglect (Vilhjalmsdottir et al. 2023). Male prisoners experienced institutionalisation, chaotic social backgrounds, mental distress (Solbakken et al. 2023), suicidal thoughts, suicide attempts, violent thoughts and acts of violence (Hemming et al. 2023). Prisoners developed negative thoughts that they attributed to imprisonment, including nearly one fifth believing nobody cares and a slightly lower proportion reporting anger and developing bitterness (Nwefoh et al. 2020). High levels of fear and anger before suicide attempts or violence were reported (Hemming et al. 2023). In one qualitative study, a gradual deterioration of mental well‐being was reported, with feelings of a profound sense of loss and emptiness (Solbakken et al. 2023), and a sense of feeling trapped in a cycle of despair was highlighted, leading to thoughts of self‐harm or suicide (Hassan et al. 2015). Prisoners described a lack of purpose and future, which exacerbated feelings of hopelessness (Hemming et al. 2023). Childhood trauma and parenting stress were often reported as the antecedents to depression and thought problems, with imprisoned mothers reporting more such issues than imprisoned fathers (Milavetz et al. 2021).

##### 3.3.1.3 Isolation

Isolation was described by prisoners as being cut‐off from meaningful interactions and relationships (Vilhjalmsdottir et al. 2023). Women with weak family ties were more likely to experience stress, although work tasks performed in prison were a protective factor (Constantino et al. 2016). Dehumanisation and loss of identity caused by isolation were described as a sense of emotional detachment (Hemming et al. 2023). Social consequences of imprisonment were described in one study, citing family embarrassment (45%) and spouses leaving (7%) and, thus, the loss of social support (Nwefoh et al. 2020). Solitary confinement resulted in psychological distress due to inactivity, social isolation and feelings of dehumanisation (Western et al. 2021).

##### 3.3.1.4 Dehumanisation

Prisoners' perception of staff as disengaged and indifferent (Fedock et al. 2024) reinforced feelings of dehumanisation. Some male detainees were reported as requiring medical attention for self‐harm injuries, but most did not receive mental health support (Clemente‐Faustino and de Guzman 2022). Studies reported prisoners feeling stripped of their humanity and unable to escape the dehumanising conditions of imprisonment (Solbakken et al. 2023). Overcrowding was described as contributing to deteriorating mental health and a lack of family visits, increasing emotional distress (Clemente‐Faustino and de Guzman 2022).

#### 3.3.2 Accessibility of Mental Health Services

Access to mental health services in prisons was limited, with several studies highlighting barriers such as a perceived lack of empathy from staff, short appointments, poor medication management and systemic barriers.

##### 3.3.2.1 Perceived Lack of Staff Empathy and Short Appointments

Prisoners perceived staff as disengaged (Fedock et al. 2024). In one study, slightly less than half (48%) were satisfied with the healthcare provided (Nwefoh et al. 2020). Fifty‐seven per cent of prisoners said staff took their negative feelings seriously or cared about their well‐being (Costa et al. 2021). Short appointments with mental health professionals left prisoners feeling that their concerns were not addressed (Fedock et al. 2024; Hassan et al. 2015), contributing to a lack of trust in the system (Vilhjalmsdottir et al. 2023). Prisoners perceived that staff lacked the necessary skills and empathy to meet their mental health needs (Cobb and Farrants 2014).

##### 3.3.2.2 Medication Management Problems

Some prisoners were prescribed antipsychotics, mood stabilisers, antidepressants, hypnotics or anxiolytics (Hassan et al. 2015). In one study, 77% of female prisoners were prescribed psychotropic medication, mainly antidepressants, whereas 51% took more than one type of psychotropic medication (Bentley and Casey 2017). Positive effects of psychotropic medication were highlighted, including managing mood, feeling stable, reducing symptoms, clearing thoughts and helping cope with imprisonment (Bentley and Casey 2017), but problems with medication management were reported as a barrier to accessing mental healthcare in prisons. Prisoners experienced changes to their prescribed medications without prior consultation or explanation, leading to distress (Hassan et al. 2015). In addition, over‐reliance on medications was highlighted (Cobb and Farrants 2014; Runte‐Geidel et al. 2014; Solbakken et al. 2023; T. Walker, Shaw, et al. 2017a). Prisoners described their experience of medication being used as a tool to control, coerce and calm them down, rather than treat their underlying mental health conditions (Cobb and Farrants 2014). Insufficient follow‐up of medication side effects was reported (Fedock et al. 2024).

##### 3.3.2.3 Systemic Barriers

Systemic barriers to mental health support reported by prisoners included inadequate staff training (Fedock et al. 2024; Hassan et al. 2015; Nwefoh et al. 2020) and insufficient resources (Fedock et al. 2024; Western et al. 2021). In one study, over 80% of prisoners reported dissatisfaction with the support provided by the prison (Antonetti et al. 2018; Nwefoh et al. 2020); unmet mental health needs were reported (Antonetti et al. 2018; Western et al. 2021). This was particularly striking in a Nigerian prison, where nearly 40% of prisoners screened positive for depression using validated instruments, but less than 10% were identified by prison health authorities as having this disorder (Nwefoh et al. 2020). Small proportions of prisoners with identifiable mental health problems receiving treatment were reported elsewhere too (Milavetz et al. 2021). Logistical challenges, rigid confinement schedules and a lack of follow‐up were problematic (Fedock et al. 2024). Prisoners felt their efforts to seek help were futile, with lack of engagement or support from mental health staff leading to feelings of abandonment (Cobb and Farrants 2014). Sometimes, prisoners did not know whom to turn to for help (Costa et al. 2021). A study conducted in England and Wales reported that although there was support available from the safer custody team, the medical response to self‐harm incidents was limited (T. Walker et al. 2021). A safer custody team is present in every prison in England and Wales, responsible for identifying and supporting prisoners at risk of self‐harm, suicide or violence and for overseeing safety processes, including the Assessment, Care in Custody and Teamwork (ACCT) framework. One study identified that better support systems were linked to fewer mental health symptoms (Milavetz et al. 2021).

#### 3.3.3 Barriers to Seeking and/or Receiving Support

Several barriers deterred prisoners from seeking mental health support, including stigma, confidentiality concerns and a perceived lack of genuine care from staff.

##### 3.3.3.1 Stigmatisation

Stigma surrounding medication use was reported as a barrier to seeking help (Bentley and Casey 2017). A prison culture of hypermasculinity was reported to reinforce the stigma associated with mental health struggles (T. Walker, Shaw, et al. 2017a). Prisoners thought expressing vulnerability would be seen as a weakness (Cobb and Farrants 2014). There were concerns about being negatively judged by both staff and fellow prisoners for seeking support (Solbakken et al. 2023).

##### 3.3.3.2 Confidentiality Concerns

Prisoners feared that their personal information would not be kept secure (Solbakken et al. 2023) or that their private information could be shared without consent (Cobb and Farrants 2014; Fedock et al. 2024), leading to a lack of trust in the mental health system. In addition, prisoners were wary of discussing sensitive issues due to fears of retaliation or exposure (Fedock et al. 2024).

##### 3.3.3.3 Perceived Lack of Genuine Care

A perceived lack of genuine care, even neglect from mental health professionals (Cobb and Farrants 2014), a slack of good relationships and trust within the prison environment (Antonetti et al. 2018), and inadequate follow‐up (Fedock et al. 2024) variously deterred prisoners from seeking support. Studies described prisoners feeling that their voices were unheard and their experiences were invalidated, contributing to a culture of mistrust and isolation (Solbakken et al. 2023). The perceived lack of genuine care also resulted from the over‐reliance on medications, seen as a means to control behaviour rather than provide therapeutic benefits and from feeling only sedated rather than symptomatically improved (Cobb and Farrants 2014); negative perceptions of medication hindered prisoners from seeking support (Solbakken et al. 2023) and led them to question the clinical judgement of doctors in prison (Hassan et al. 2015).

#### 3.3.4 Coping Strategies

In response to the challenges faced, prisoners employed various coping strategies to manage their mental health conditions. These included adaptive strategies such as adhering to prison rules, peer support, religious practices and engagement in meaningful activities and maladaptive strategies, including self‐harm, substance use and avoidance strategies.

##### 3.3.4.1 Adaptive Strategies

Studies reported prisoners *adhering to institutional rules* to avoid punishment (Clemente‐Faustino and de Guzman 2022), while using therapeutic techniques learned during therapy sessions, such as revisiting past communications with therapists, to maintain psychological stability (T. Walker, Shaw, et al. 2017a). *Peer* *support* was a coping strategy. Some prisoners preferred self‐reliance or seeking support from family or partners rather than formal prison support structures (Costa et al. 2021), but other studies reported prisoners forming strong bonds with each other, offering mutual support during times of personal loss or emotional distress (Cobb and Farrants 2014). Peer support was highlighted as central to fostering a sense of community and reducing feelings of isolation (T. Walker, Shaw, et al. 2017a) and providing a sense of purpose and belonging, helping prisoners navigate the challenges of prison life (Clemente‐Faustino and de Guzman 2022). Social interaction was reported to help reduce feelings of isolation and foster a sense of belonging (Clemente‐Faustino and de Guzman 2022). *Religious practices* and activities were reported as providing a sense of hope, purpose and emotional support and fostering a sense of community (Clemente‐Faustino and de Guzman 2022). In one study, over 80% reported becoming more religious to cope with imprisonment (Nwefoh et al. 2020). Some engaged in *activities*, such as reading or writing, to maintain a sense of purpose and cope with the stresses of imprisonment (T. Walker, Shaw, et al. 2017a), creative expression (Fedock et al. 2024) or reading, avoiding negativity and staying in therapy (Maruca et al. 2017) as a means of coping with the stresses of imprisonment. A preference for nonmedical management and social interactions was reported, including “someone to talk to” (Solbakken et al. 2023).

##### 3.3.4.2 Maladaptive Strategies

High levels of emotional distress were linked to *self‐harm* (T. Walker et al. 2021), with just over half of prisoners reporting on this using self‐harm for the release of or relief from emotions, just over 10% for coping and calming and 5% or less for gaining control, expression of suicidal ideation, escapism and dissociation from the environment due to lack of function or uncertainty or to manage the influence of voices (T. Walker et al. 2021). This behaviour was identified as cyclical, exacerbated by the isolating prison environment, which fostered negative thoughts and emotions (Solbakken et al. 2023). Such reports occurred among both women (T. Walker et al. 2021) and men (Clemente‐Faustino and de Guzman 2022). Some prisoners with self‐harming behaviours were provided brief psychodynamic interpersonal therapy (T. Walker, Shaw, et al. 2017a). A lack of effective support systems was seen as contributing to the prevalence of self‐harm, with prisoners feeling that their needs for adequate mental healthcare was unfulfilled (Solbakken et al. 2023).

High levels of *substance abuse* were highlighted as a means of coping (Antonetti et al. 2018; Rose and LeBel 2020; Vilhjalmsdottir et al. 2023). Prisoners used drugs to mask or forget their problems (Vilhjalmsdottir et al. 2023) and misused prescribed medications, attributed to a lack of sufficient mental healthcare to provide immediate relief from suffering (Clemente‐Faustino and de Guzman 2022). *Avoidance strategies* used by prisoners to temporarily escape the stress of imprisonment included avoiding the emotional processing of their experiences, attributed to a perceived lack of support within the prison system (Cobb and Farrants 2014). The lack of follow‐up and perceived lack of empathy from mental health professionals contributed to the perception that seeking help was futile (Fedock et al. 2024).

## 4 Discussion

This review synthesised evidence from 19 studies about prisoners’ mental health, illness and its treatment and their care within the prison environment. Four key themes emerged from the systematic search and narrative synthesis: (1) emotions and feelings, (2) accessibility of mental health services, (3) barriers to seeking and/or receiving support and (4) coping strategies.

In our review, prisoners reported a wide range of emotional suffering—from severe mental illness through experiences of despair, hopelessness and isolation to maladaptive coping mechanisms of self‐harm. These findings are mirrored in the wider literature. For example, a systematic review exploring PTSD in prison settings (and therefore excluded from this review, which was restricted to primary studies) found associations between the severity of the condition and behaviours such as self‐harm and aggression (Facer‐Irwin et al. 2019). Our review identified repeated evidence of prisoners feeling dehumanised, in part by the treatment available. A study involving American released prisoners, sampled from community mental health settings (and therefore outside this review’s inclusion criteria, which were restricted to studies conducted within prison settings), seems to endorse experiences reported while still in prison; they recalled “rationing” of care, or being dismissed, leading them to believe that prisoners’ lives do not matter (Canada et al. 2022). The gap between the need for mental health support and the availability of services in prison was evident in all studies included in our review. There may be an important distinction to be made here between recognition and failure to provide care and never recognising a treatable problem. One of our included studies, for example, found that although nearly 40% of prisoners screened positive for depression using validated instruments, only 7% were identified by prison health authorities as having this disorder (Nwefoh et al. 2020). Specific ‘treatment gaps’ also exist; one study, for example, found that 90% of those with a PTSD diagnosis in prisons are not receiving treatment for it (Bebbington et al. 2017). The need‐care deficit is also evident in accessing services outwith the prison setting. The average waiting time for transfer from prison to hospital for urgent treatment in England and Wales is 85 days, substantially exceeding the 28‐day standard set, with only 15% of patients transferred within this timeframe (Centre for Mental Health 2025; HM Chief Inspector of Prisons 2024). This delay suggests systematic failures at the interface between healthcare delivery in the prison and the wider community. In 2006, in the United Kingdom, healthcare delivery in prisons was transferred from the Home Office to the Department of Health, and services are commissioned from healthcare providers; healthcare staff are not employed by the prison system. This transfer was made to ensure that prisoners received optimal healthcare at the point of need, but this is not working well. Reasons may include understaffing in both prisons and health services, and independent bodies are clear about substantial shortfalls in the nature and extent of health service delivery to prisoners (Centre for Mental Health 2025; HM Chief Inspector of Prisons 2024). Elsewhere, evidence suggests access to services is hindered by cumbersome referral procedures and inadequate staffing (Canada et al. 2022). In our review, prisoners said the default response from staff was disbelief about their medical and mental health complaints, with suggestions that prisoners would fake ailments for material gain or attention (Canada et al. 2022).

Our included studies suggest that, for some prisoners, mental health problems may be worsened, and sometimes caused, by the prison setting itself, perhaps through traumatising and re‐traumatising individuals, though evidence on the overall trajectory of mental health during imprisonment remains mixed. It is important, however, to acknowledge that the relationship between imprisonment and mental health is not straightforwardly negative. A systematic review of longitudinal studies of mental state among people over time in prison did not find any systematic evidence of deterioration but, rather, allowing for such problems peaking around prison entry, a tendency to improve (J. Walker et al. 2014). One study included in this review, for example, tracked prisoners with psychosis over 12 weeks and found that psychotic symptoms decreased over time for the largest subgroup, proposing that the structure and routine of the prison environment may have a stabilising effect for some individuals (Blaauw et al. 2007). A longitudinal study of life‐sentenced prisoners in England and Wales described sedative coping and how, over time, prisoners reported that the outside world became progressively irrelevant, whereas the prison cell became a primary source of emotional security and control (Crewe 2024). Prison itself will undoubtedly have an impact on staff and prisoners alike; the crucial objective is to minimise harm and maximise opportunities for the prevention or treatment of mental disorders.

A recent review exploring the role and impact of prison chaplains found that they provide pastoral and emotional support as well as religious, practical and educational input, with impacts including rehabilitation, creation of communities, calm, forgiveness and atonement (Jarrett et al. 2024). Prisoners described chaplains as nonjudgemental, trustworthy and genuinely caring. This contrasted with descriptions by participants in studies in our review describing mental health staff as coercive, controlling,and focused on behavioural management. Although chaplaincy services cannot substitute for mental healthcare, they may provide a conduit or a model for enhancing practice. This review suggests that chaplains operate within a framework of unconditional positive regard and spiritual care, potentially creating relational conditions that may facilitate prisoners’ willingness to engage. There was some evidence in the review that prisoners reported preferring chaplain support over, say, the prison psychologist, as chaplains did not ask questions; they simply listened. This suggests that the barrier to help‐seeking may not be an inherent reluctance on prisoners’ part but rather some specific features of formal mental health examinations that prisoners find problematic. Among prisoners who engaged with religious services and pastoral support, reasons for doing so included enjoyment of singing; as a way of seeking peace, seeking forgiveness and dealing with the drudgery of prison life, social contact; and as a way of getting out of their cells and having a coffee, but they also specifically mentioned support for mental health and well‐being. This suggests a need to explore ways to provide or enhance existing spiritual frameworks within prison settings, including the role of the prison chaplain in linking prisoners with mental health services when appropriate.

### 4.1 Strengths and Limitations

A comprehensive and robust search strategy was implemented, including backward and forward citation tracking; however, it is not possible to guarantee that we captured all relevant empirical research on this topic. With respect to the included papers, most were conducted in high‐income countries, and there was a scarcity of research addressing this topic globally. In addition, as searches were restricted to English‐language studies, insights from regions where prison practices and contexts differ may not be fully represented in this review. A further limitation is that the included studies did not distinguish between prisoners who were seeking mental health support and those already in receipt of it. Although our review question included both groups, the literature did not draw a boundary between them, and the relevant included studies reported only on prisoners who needed, but were not receiving, formal support. Future studies should differentiate between help‐seeking and help‐receiving populations to provide more nuanced findings. Furthermore, there is tension between depth and breadth in this area of research. The predominantly qualitative and small‐sample studies included here offer rich, detailed insights into the experiences of prisoners living with mental health conditions, but the findings may not be generalisable across wider prison populations. Nevertheless, such depth of qualitative data is invaluable for developing more nuanced and meaningful questions for future research.

### 4.2 Recommendations for Future Research

Research exploring ways of reducing limitations and enhancing opportunities in prison mental healthcare delivery is, thus, indicated—not only directly with qualified healthcare practitioners and for diagnosed mental health problems but also through networks with pastoral support for optimising links and preventive strategies. The role of anti‐stigma campaigns and work to normalise help‐seeking should be explored and evaluated. Exploring ways of developing prosocial prisoner peer support networks could reduce self‐harm. The Listener scheme, a Samaritans‐supported initiative operating across almost every prison in England and Wales, provides a model in which trained prisoners offer peer support to those in distress, with evidence of benefit for both recipients and Listeners themselves (Samaritans 2026).

Future research should also examine prison staff roles that are expected to contribute to prisoner mental well‐being. Under the Offender Management in Custody (OMiC) model, for example, prison officers are key workers tasked with developing constructive, motivational relationships with prisoners (Ministry of Justice 2026), yet the mental health dimensions of this role remain under‐evaluated. The Assessment, Care in Custody and Teamwork (ACCT) framework similarly requires multidisciplinary collaboration across all staff groups in supporting prisoners at risk of self‐harm or suicide (Ministry of Justice 2026). Psychologically Informed Planned Environments (PIPEs), a further model, which as the part of the NHS England and HMPPS Offender Personality Disorder pathway, offer a further model that trains staff to engage in a psychologically informed, relationally focused way, with preliminary evidence suggesting improvements in social functioning and reductions in interpersonal behaviour (Freestone et al. 2024). Prison‐based therapeutic communities similarly offer transferable lessons, particularly around the value of structured, relationally rich environments and peer engagement in supporting those with complex mental health needs (Richardson and Zini 2021). Future research should seek to better understand the factors that determine whether mental health deteriorates or improves during imprisonment, including sentence length, access to support and pre‐existing conditions.

### 4.3 Recommendations for Practice

The findings of this review have direct implications for practice. Prisoners with mental health conditions face a dual burden, including the psychological weight of imprisonment and barriers to accessing appropriate support, including stigma, distrust of services and institutional obstacles. Critically, this review highlights that what works already exists in many cases, with peer support models such as the Listener scheme, psychologically informed environments through PIPEs and multidisciplinary frameworks such as ACCT, yet implementation remains inconsistent and access inequitable. The priority for practice, therefore, is not the development of new protocols but the enabling of wider, more consistent implementation of evidence‐informed approaches that are already available.

Frontline prison staff are key in this regard. Given that prisoners described distrust of formal services and reluctance to disclose mental health difficulties, the quality of everyday relational interactions between staff and prisoners may be as important as formal clinical intervention. Training and supporting nonclinical staff, including prison officers, chaplains and peer supporters, to respond to mental distress in a psychologically informed way should be a practical priority. Anti‐stigma work aimed at the prison environment, addressing both prisoner and staff attitudes towards mental health help‐seeking, represents a further area for practical consideration.

## 5 Conclusion

Our review focuses on evidence about the experiences of prisoners living with mental health conditions and their experiences of mental health support in prison; the high prevalence of mental disorders among prisoners is already well established. Prisoners commonly recognise at least some of their needs in this respect, but there is evidence that they have little confidence in getting help from mental health services. This means they are commonly reluctant to seek formal mental health support. Many behaviours, such as self‐harm or substance use in prison, are forms of maladaptive self‐management. More positive alternatives include responses to faith leaders and to ‘official’ peer support, such as Listeners. Future research should focus on how to optimise these in themselves and as bridges to specific treatment when needed. There would also be benefit in evaluation of prison officer roles in this as explicit schemes, such as personal PIPEs, trauma‐informed approaches and pathway work, develop. For practice, the central imperative is not the revision of existing protocols but the equitable implementation of what the evidence already shows works, including psychologically informed, relationally grounded and peer‐supported approaches to mental health in prison.

## Author Contributions

All six authors (A.K., J.B., J.D., D.B., J.S. and M.J.) conceived the idea for the review, methods were designed by A.K., J.B. and J.D. A.K. extracted and curated data. A.K. drafted the manuscript, and all named authors revised and approved the final version. All authors agree to be personally accountable for the accuracy and integrity of the work.

## Funding

The corresponding author received doctoral research funding from Birmingham City University. The funding is a doctoral research or training grant. The findings of this review will contribute to the thesis.

## Ethics Statement

The authors have nothing to report.

## Consent

The authors have nothing to report.

## Conflicts of Interest

The authors declare no conflicts of interest.

## Data Availability

Data sharing is snot applicable to this article as no datasets were generated or analysed during the current study.
